# Improved visibility of character conflicts in quasi-median networks with the EMPOP NETWORK software

**DOI:** 10.3325/cmj.2014.55.115

**Published:** 2014-04

**Authors:** Bettina Zimmermann, Alexander W. Röck, Arne Dür, Walther Parson

**Affiliations:** 1 Institute of Legal Medicine, Innsbruck Medical University, Innsbruck, Austria; 2 Institute of Mathematics, University of Innsbruck, Innsbruck, Austria; 3 Penn State Eberly College of Science, University Park, PA, USA

## Abstract

**Aim:**

To provide a valuable tool for graphical representation of mitochondrial DNA (mtDNA) data that enables visual emphasis on complex substructures within the network to highlight possible ambiguities and errors.

**Method:**

We applied the new NETWORK graphical user interface, available via EMPOP (European DNA Profiling Group Mitochondrial DNA Population Database; *www.empop.org*) by means of two mtDNA data sets that were submitted for quality control.

**Results:**

The quasi-median network torsi of the two data sets resulted in complex reticulations, suggesting ambiguous data. To check the corresponding raw data, accountable nodes and connecting branches of the network could be identified by highlighting induced subgraphs with concurrent dimming of their complements. This is achieved by accentuating the relevant substructures in the network: mouse clicking on a node displays a list of all mtDNA haplotypes included in that node; the selection of a branch specifies the mutation(s) connecting two nodes. It is indicated to evaluate these mutations by means of the raw data.

**Conclusion:**

Inspection of the raw data confirmed the presence of phantom mutations due to suboptimal electrophoresis conditions and data misinterpretation. The network software proved to be a powerful tool to highlight problematic data and guide quality control of mtDNA data tables.

It has been observed that the generation of mitochondrial (mt)DNA (population) data are prone to error (1-4). A valuable tool for graphical representation of mtDNA data is quasi-median network (QMN) construction of reduced and filtered haplotypes (1). Clerical errors, sequencing artifacts, and other ambiguous data may induce character conflicts that increase the complexity of the network, pinpointing initial points of action for quality control of mtDNA data sets (1-4). This tool is provided via the EMPOP database, a collaborative project for the provision of high-quality mtDNA population data for forensic purposes, which was initiated by the European DNA Profiling Group (EDNAP; *http://www.isfg.org/ednap*)**in 1999. The acronym stands for “EDNAP mtDNA population database” and despite of its primary purpose of providing reliable frequency estimates, the website (*www.empop.org*) has regularly been used for quality control (QC) of published and newly submitted population data (3,4). QMNs form one part of the QC concept performed by EMPOP when mtDNA population data are submitted for publication in *Forensic Science International Genetics* (5) and *International Journal of Legal Medicine* (6) and thus contribute to the quality improvement of published mtDNA data sets. Also, all haplotypes presented in the mtDNA database EMPOP (3) undergo rigorous quality control prior to upload. This procedure has proven to be successful in detecting errors in individual data sets and collaborative exercises (4,7,8).

While the calculation and the drawing of QMNs is supported by software (NETWORK) freely accessible via the EMPOP website, its successful interpretation and evaluation depends on the experience of the user. Users have brought to our attention that QMNs generated by NETWORK are sometimes too complex and fraught with reticulations, rendering the identification of potential errors difficult. In particular, data sets of large sample sizes (>500) were concerned, as well as data harboring haplotypes from distant phylogenies (eg, South American populations including haplogroup L, M, and N lineages).

In this study, we describe the application of a new graphical user interface (GUI) of the NETWORK tool that offers the possibility to visually highlight selected structures within the graph for a better distinction of reticulations in complex areas (9). Further, haplotypes are now directly linked to the graphical representation of the nodes and can be examined in a convenient way to identify potential errors such as phantom mutations, clerical errors, violation of alignment rules, and artificial recombination. The performance and features of the new GUI are demonstrated by example of two data sets submitted to EMPOP QC.

## Material and methods

The study took place at the Institute of Legal Medicine, Innsbruck Medical University, during summer 2012. The application of the new NETWORK GUI was demonstrated by two mtDNA population data sets that were submitted for QC. The data sets are kept anonymous and comprised 320 mtDNA haplotypes from West Eurasia (data set A) and 230 haplotypes from East Asia (data set B). QMN analysis was conducted using EMPOP NETWORK as outlined earlier (4). The removal of rapidly evolving mutations is critical for the readability of QMNs. The user can choose between different types of filters depending on the application (4). Here, the data sets were filtered with *EMPOPall_R11*, which removed all mutations observed and documented by raw lane data in that respective EMPOP release (Release 11) (3). Thus, only newly observed differences to the revised Cambridge Reference Sequence (10) remained in the network, which provides a first overview of the data quality. Authors were contacted after EMPOP QC and asked to submit raw data of the haplotypes in question to evaluate the QMN findings.

## Results

MtDNA population data sets, as well as individual mtDNA haplotypes, can be quality controlled using the freely accessible EMPOP NETWORK tool. This procedure involves two consecutive steps: first, all haplotypes undergo plausibility checks. The rCRS-coded haplotypes are checked for plausibility, eg, with regard to sequence range violation (eg, T489C in a defined range of 73-340), reference bias (eg, A263A), double specification of mutations, and wrong notations of insertions and deletions. We have demonstrated earlier that many errors are already unmasked at this stage (4). Second, quasi-medians are calculated based on the settings selected by the user. QMN analysis involves the application of filters to remove highly recurrent mutations (*EMPOPspeedy*) that would otherwise lead to complex structures in the network and reduce its readability. The most comprehensive filter includes all documented differences to the rCRS (*EMPOPall)* (3), which reduces the complexity of the network to new observations. We recommend using this filter as a first indicator of the quality of an mtDNA data set, as it provides a first overview on unobserved mutations. The current EMPOP release (R11) holds 1694 documented differences at 1073 positions within the control region and includes a large portion of known lineages.

**Figure 1:**
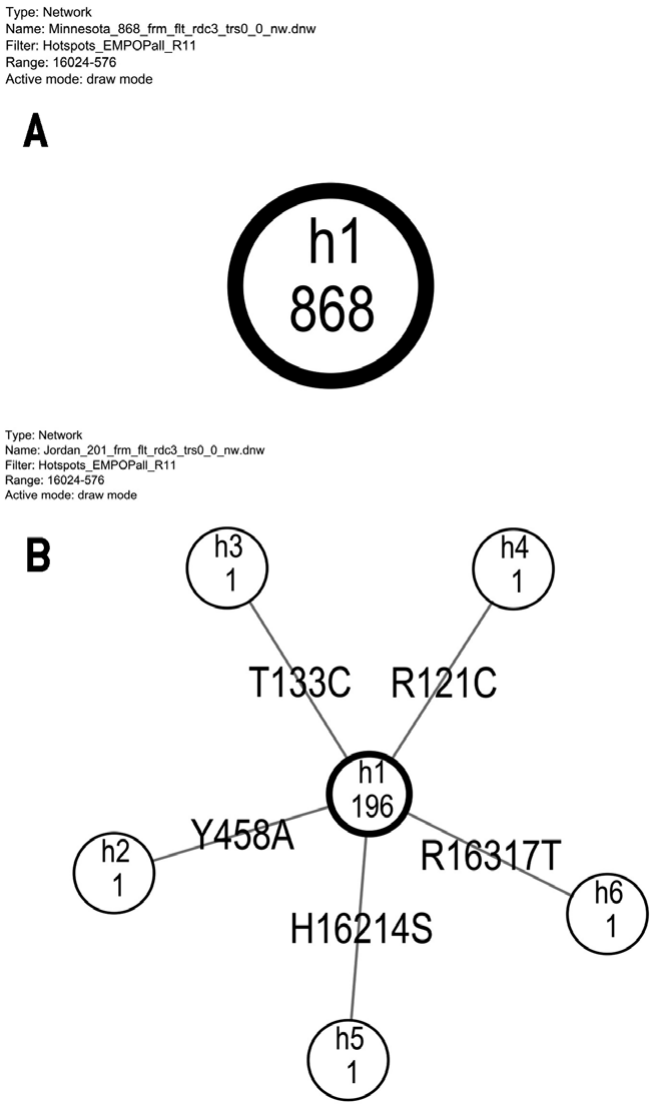
Quasi-median networks (QMNs) generated from (**A**) 868 haplotypes from Minnesota already included in EMPOP (accession numbers EMP00402-EMP00406) and from (**B**) 201 haplotypes from Jordan (submitted to EMPOP for quality control). For both data sets the *EMPOPall_R11* filter including all differences to the rCRS observed in EMPOP Release 11 was applied. Thus, data sets already included in EMPOP collapse into a single node (**A**). Data sets not yet included in EMPOP produce structures that are reduced to the newly observed differences to the rCRS (**B**). Here the QMN shows a simple star-like structure displaying five polymorphisms not yet observed in EMPOP. The branch labeled “H16214S” for example represents a point heteroplasmy at position 16214 in haplotype h5 with haplogroup status D4i. This observation was confirmed by the raw data (Supplementary Figure 1) (web extra material 1).

Its application to data already included in EMPOP results in the QMN of a single node as all annotated differences to the rCRS in that data set were filtered (Figure 1A). MtDNA data from already sampled populations (eg, Westeurasian populations) that were generated under forensic guidelines (11-13) typically result in simple QMN torsi after passage through the *EMPOPall* filter (Figure 1B) as only few novel differences to the rCRS are observed. These can then be evaluated by the raw lane data (Supplementary Figure 1) (web extra material 1), which in this case confirmed all observations. We note, that new lineages are continuously observed especially in cases where remote populations were sampled. These then leave their haplotypic signatures in the QMNs.

### QMN analysis of data set A

**Figure 2:**
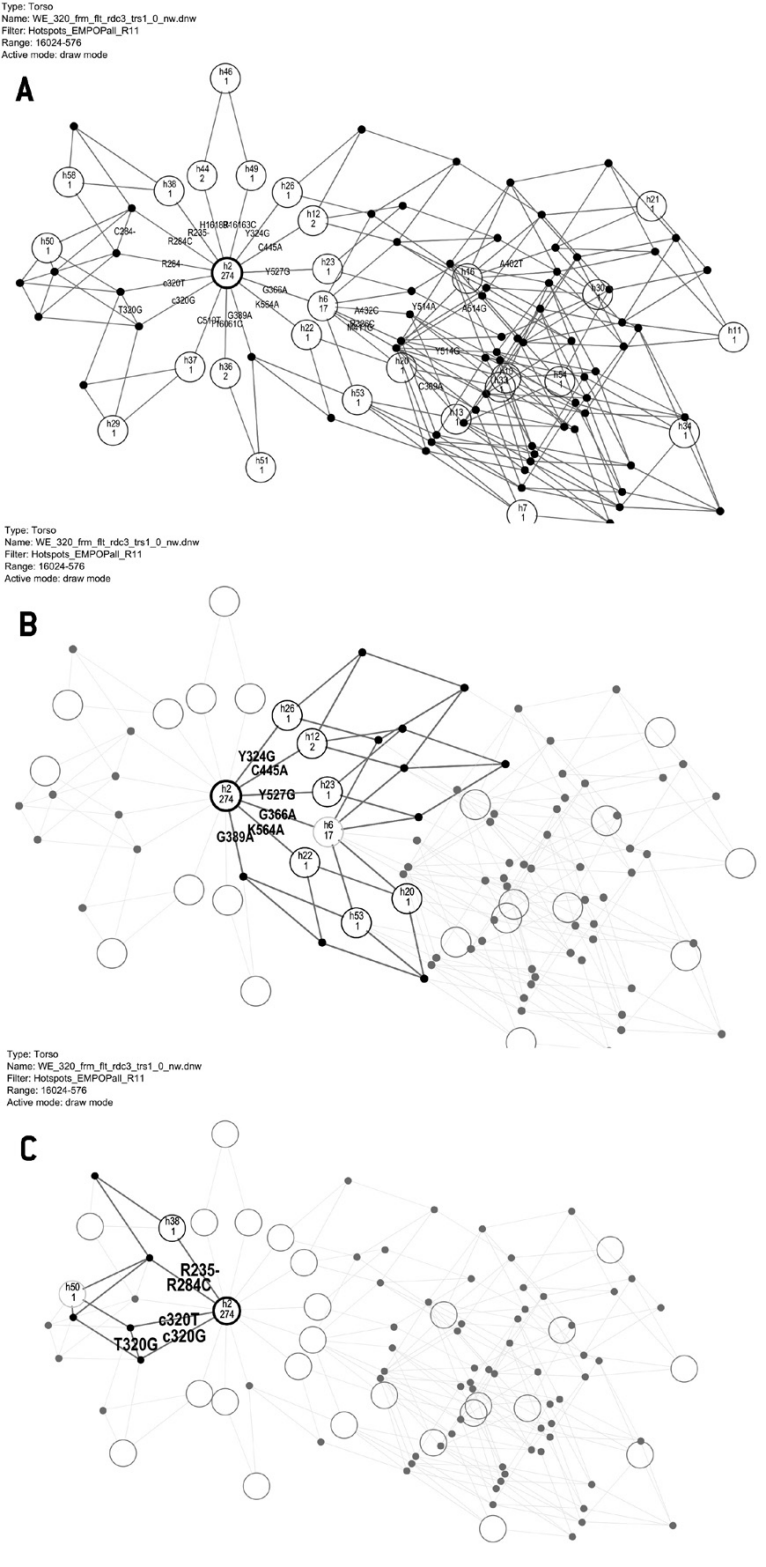
Quasi-median networks (QMN) torso of 320 mtDNA haplotypes from a West Eurasian population sample after passage through the *EMPOPall* (Release 11) filter (**A**). The complexity of the torso is caused by mutations that were not observed in EMPOP Release 11. (**B**) The accentuated sub-graph of the QMN torso. Node h6 was selected by mouse-click. This node together with node h20 included 18 haplotypes that all carry mutation A366G. (**C**) The accentuated sub-graph of the QMN torso selecting node h50 (branch R284C). The linking branches c320T and c320G represent phantom mutations that are also caused by length heteroplasmy in the HVS-2 C-tract.

The calculation of the QMN of data set A comprising 320 haplotypes of west Eurasian provenance with the *EMPOPall_R11* filter resulted in a complex QMN torso (Figure 2A; see Figure 1B as contrast). A user would be interested in identifying those branches that cause the complex structures as they represent yet unobserved mutations that may be erroneous. Using mouse over the new GUI allows for the visual accentuation of linked nodes and branches, while the complementary substructure is dimmed. Once a subtree of interest has been identified, individual nodes can be specifically selected by mouse-click to view all haplotypes that are included in that node and thus share the difference to the rCRS indicated by the branch (Supplementary Figure 2) (web extra material 2). When evaluating QMNs filtered with *EMPOPall* it is recommended to start reviewing abundant mutations. For example, the QMN torso contained 18 haplotypes that shared A366G (nodes h6 and h20, Figure 2B). The selection of node h6 by mouse-click resulted in a list of the 17 affected haplotypes (Supplementary Figure 2) (web extra material 2). The high abundance of A366G in various haplogroups (R0, H15, H2a2b, H6, HV0, J1c2, and K1a) was surprising and worth inspecting the respective raw data. This review clearly indicated the presence of a phantom mutation at position 366 due to overlaid sequence electropherograms originating from length heteroplasmy in the HVS-2 C-tract around position 309 (Supplementary Figure 3) (web extra material 3). The adenine bases 5 prime of position 366 were shifted downstream and masked the G signal at position 366. Additional reverse sequencing reactions would help calling the correct variant. This first part of the QMN review already suggested that only single stranded sequencing information had been used to generate the reported consensus haplotypes, which does not meet the recommendations in forensic genetics (11,12). These findings are confirmed by other phantom mutations in HVS-2 downstream of the C-tract, eg, c320T, c320G (Figure 2C). These (and other) errors in this data set have been reported as hot-spots for phantom mutations earlier (14).

### QMN analysis of data set B

**Figure 3:**
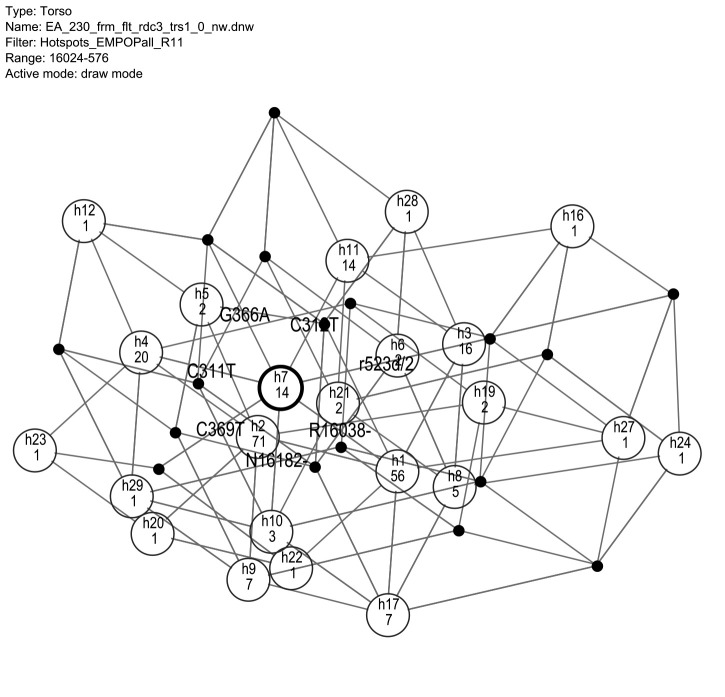
Quasi-median networks (QMN) torso of 230 mtDNA haplotypes from an East Asian population sample after passage through the *EMPOPall* (Release 11) filter. Phantom mutation G366A previously discussed for data set A (Figure 2B) is also observed in this data set.

Sequencing problems similar to those reported in data set A were also visible in data set B, an East Asian population sample including 230 haplotypes (eg, phantom mutation at position 366, Figure 3). More worrying was the persistent deletion at position 16 038, which was reported in 173 instances (75% of all samples). Selecting the branch that carries the deletion at 16 038 did not change the appearance of the entire network, because of the enormous number of affected erroneous haplotypes. The sequence raw data suggested that the analysis suffered from electrophoretic mobility problems, which is why the two A signals at positions 16 038 and 16 039 merged into one single broad peak (Supplementary Figure 4) (web extra material 4). Another eye-catching observation was the frequent occurrence of C311T (n = 106, 46%, Supplementary Figure 5) (web extra material 5), which was absent in all EMPOP data collected so far. In contrast, the expected insertion 315.1C was missing in those cases, suggesting that this part of the HVS-2 C-tract was not reported in 3′ convention, as laid down in the forensic genetic recommendations (11).

## Discussion

The graphical representation of an mtDNA data set as QMN is a valuable tool for inspecting haplotypes and mutations that would otherwise be difficult to decipher in a tabular list. As detailed elsewhere (2,15), recurrent mutations need to be filtered and haplotypes reduced to the relevant information to decrease the complexity of the QMN and make it readable for the human eye. We explicitly note here that any interpretation of the data quality by QMN can only refer to those mutations that remain in the reduced data set. This is why QMN forms only one – albeit important – part of mtDNA data quality review. QMN analysis can be performed via the EMPOP website. Based on user feedback, we here presented an improved and updated version of this tool and demonstrated its utility using two data sets submitted to EMPOP for review.

The new network editor software presents features that considerably improve the power of quasi-median networking for data quality control. The main advantage is the possibility to accentuate subgraphs while the remaining network (complement of the induced subgraph) is dimmed. All nodes and branches causing the increased complexity become better visible. For convenient identification of the corresponding haplotypes, sample identifiers are listed upon selection of a node with the mouse. The sequence electropherograms of these samples should be examined with great scrutiny and appropriate actions taken (eg, correction of base calls, repetition of sequencing reactions with alternative primers, etc). Further practical applications included in the new network editor GUI are adjustable drawing and camera settings, with which nodes and branches can be adapted in color, size, font settings, and other. Single nodes and branches can be moved to change the structure and thus the visibility of the graph. Branches representing identical mutations stay parallel. Supported export formats include GIF, SVG, and EPS.

MtDNA data have been quality reviewed with EMPOP NETWORK since 2006, and since 2010 the journals *Forensic Science International Genetics* (11) and *International Journal of Legal Medicine* (6) have required authors to have their mtDNA data quality controlled by EMPOP prior to submission of the manuscript to the journal. It is our experience that more than half of the submissions require substantial changes due to data idiosyncrasies. The forensic community is particularly sensitive to quality issues. Nevertheless, several calls for increased quality in forensic genetics (16,17) have been ignored. With a move to massively parallel sequencing technologies the problem will likely acerbate (18), as the increased amount of sequence data likely contains more artifacts than Sanger-type sequence data, and the application of diverse alignment algorithms significantly affects the sequence coverage and thus the resulting consensus sequences (19). Powerful tools for data review and QC will become indispensable.
