# Supplementary material for: Improved visibility of character conflicts in quasi-median networks with the EMPOP NETWORK software
Source: Croat Med J. 2014 Apr;55(2):115–20. doi: 10.3325/cmj.2014.55.115 (PMC4020147; doi:10.3325/cmj.2014.55.115)

Supplementary Figure 1. The electropherograms of haplotype h5 covering position 16214. The point heteroplasmic mixture of C and G nucleotides at this position is clearly visible.

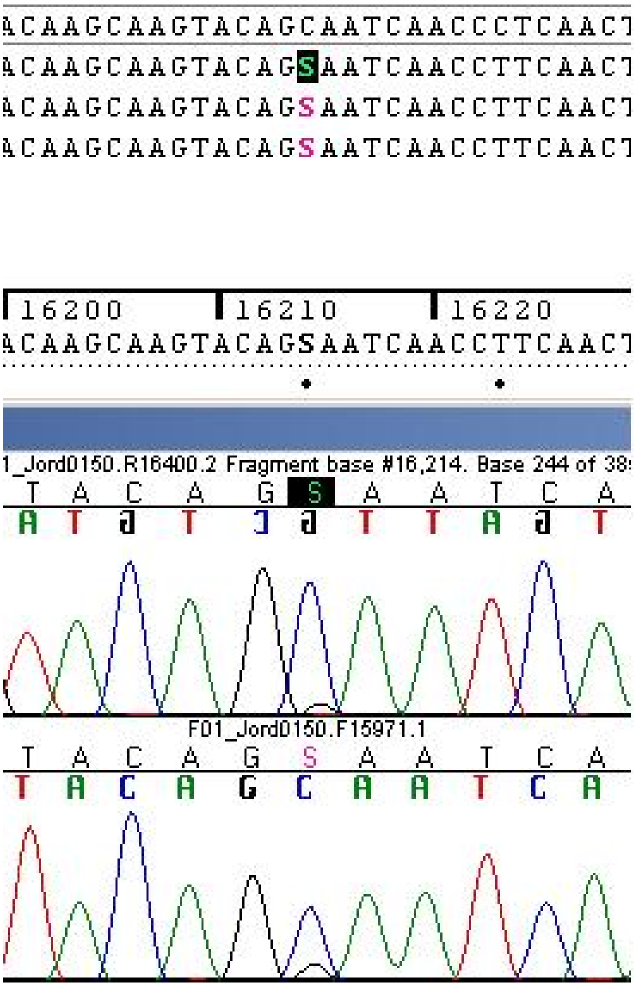

Supplement: Supplementary Figure 1 [file CroatMedJ_55_s001.pdf]
