# Supplementary material for: Improved visibility of character conflicts in quasi-median networks with the EMPOP NETWORK software
Source: Croat Med J. 2014 Apr;55(2):115–20. doi: 10.3325/cmj.2014.55.115 (PMC4020147; doi:10.3325/cmj.2014.55.115)

Supplementary Figure 2. Detailed information for the 17 haplotypes represented by node h6 (highlighted). Note that the difference at position 366 is reported for various haplogroups (hgs R0, H15, H2a2b, H6, HV0, J1c2 and K1a).

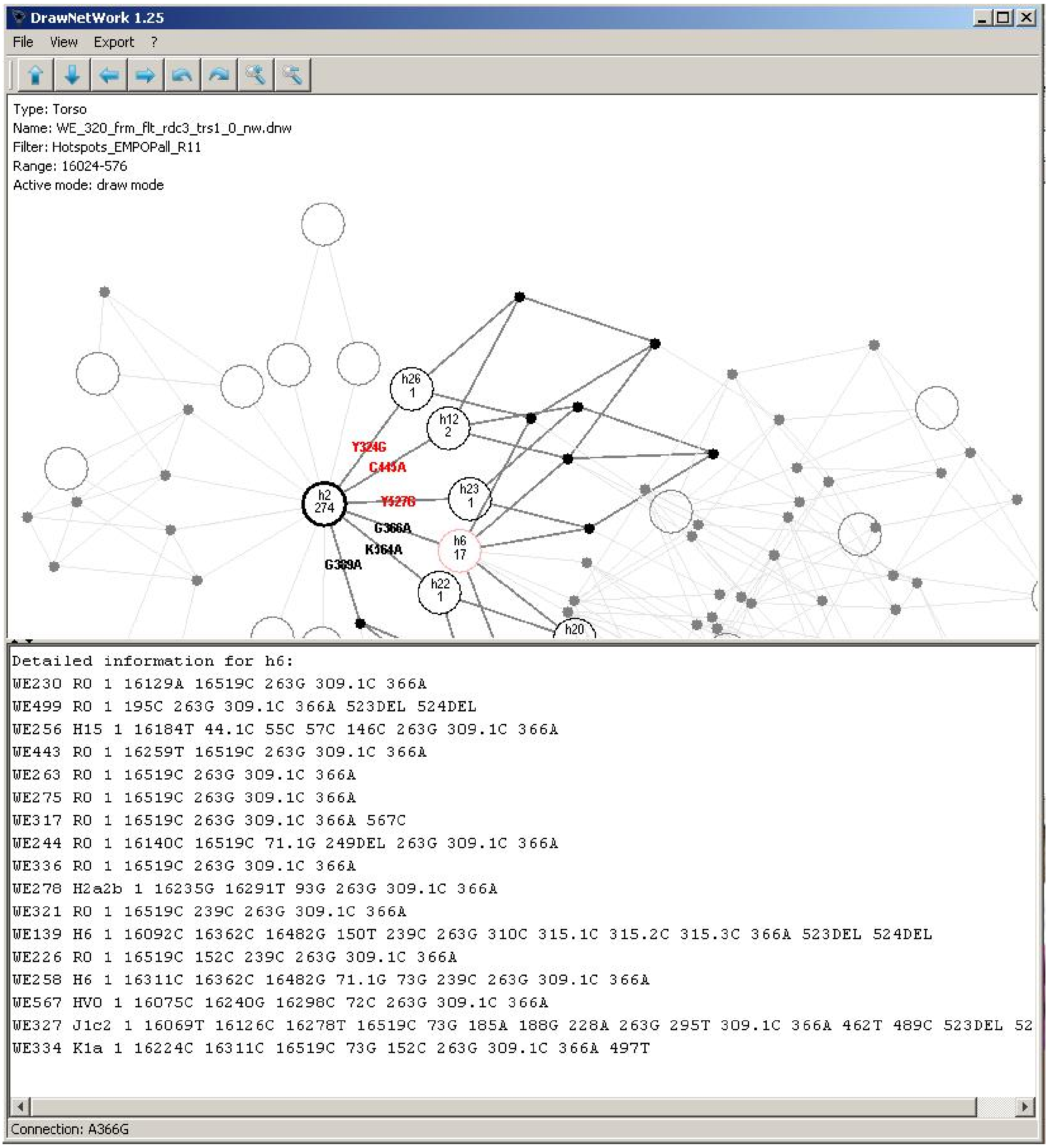

Supplement: Supplementary Figure 2 [file CroatMedJ_55_s002.pdf]
