# Supplementary material for: Improved visibility of character conflicts in quasi-median networks with the EMPOP NETWORK software
Source: Croat Med J. 2014 Apr;55(2):115–20. doi: 10.3325/cmj.2014.55.115 (PMC4020147; doi:10.3325/cmj.2014.55.115)

Supplementary Figure 3. The sequence electropherograms from position 299 to 372. Length heteroplasmy in the C-tract caused overlaid signals further downstream that were not correctly interpreted by the software and apparently taken at face value. The phantom mutation A366G remained in the data set.

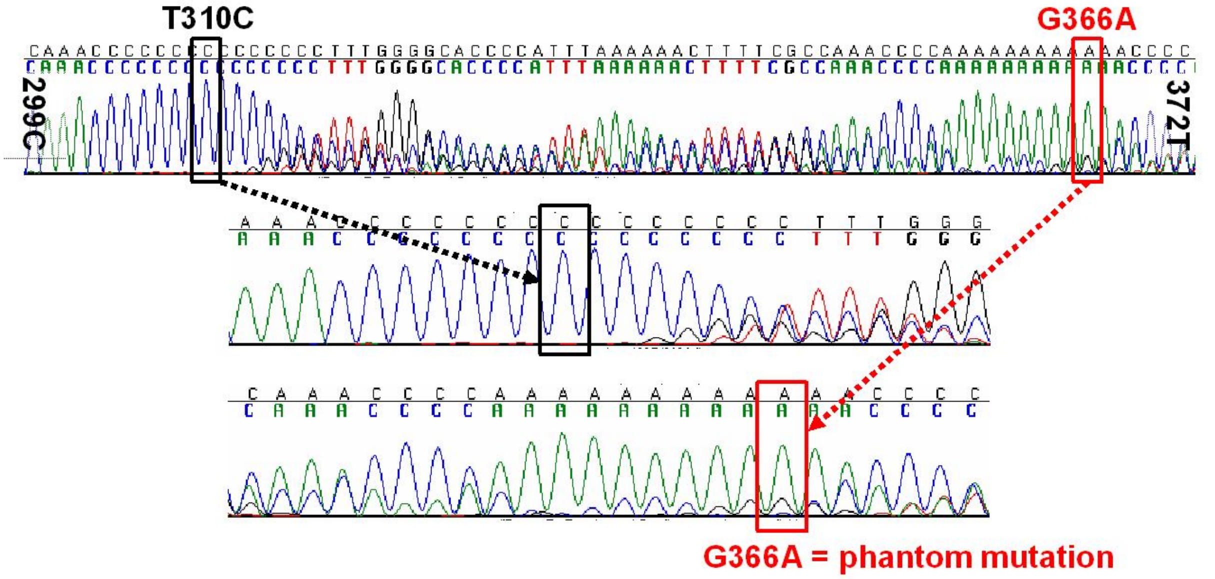

Supplement: Supplementary Figure 3 [file CroatMedJ_55_s003.pdf]
