# Supplementary material for: Improved visibility of character conflicts in quasi-median networks with the EMPOP NETWORK software
Source: Croat Med J. 2014 Apr;55(2):115–20. doi: 10.3325/cmj.2014.55.115 (PMC4020147; doi:10.3325/cmj.2014.55.115)

Supplementary Figure 4. The sequence electropherograms clearly show the origin of the phantom deletion at position 16038. Apparently the electrophoretic mobility was set too fast, which is why the two A signals at positions 16038 and 16039 merged into one broad peak. Alternative sequencing strands were lacking that could have aided correct sequence interpretation.

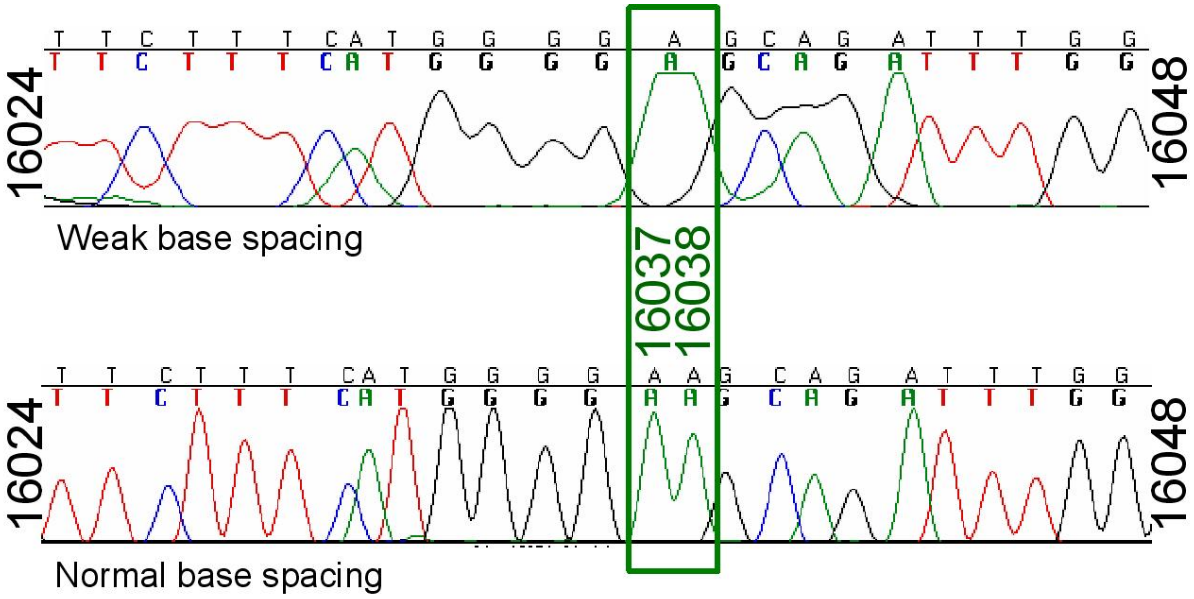

Supplement: Supplementary Figure 4 [file CroatMedJ_55_s004.pdf]
