# Supplementary material for: Improved visibility of character conflicts in quasi-median networks with the EMPOP NETWORK software
Source: Croat Med J. 2014 Apr;55(2):115–20. doi: 10.3325/cmj.2014.55.115 (PMC4020147; doi:10.3325/cmj.2014.55.115)

Supplementary Figure 5. The accentuated sub-graph of the QMN torso displayed in Figure 3A selecting node h2 that includes 71 haplotypes that harbor the deletion at position 16038. The linking mutation 311T, present in 46% of the data, originates from the 5’ alignment in the HVS-2 C-tract.

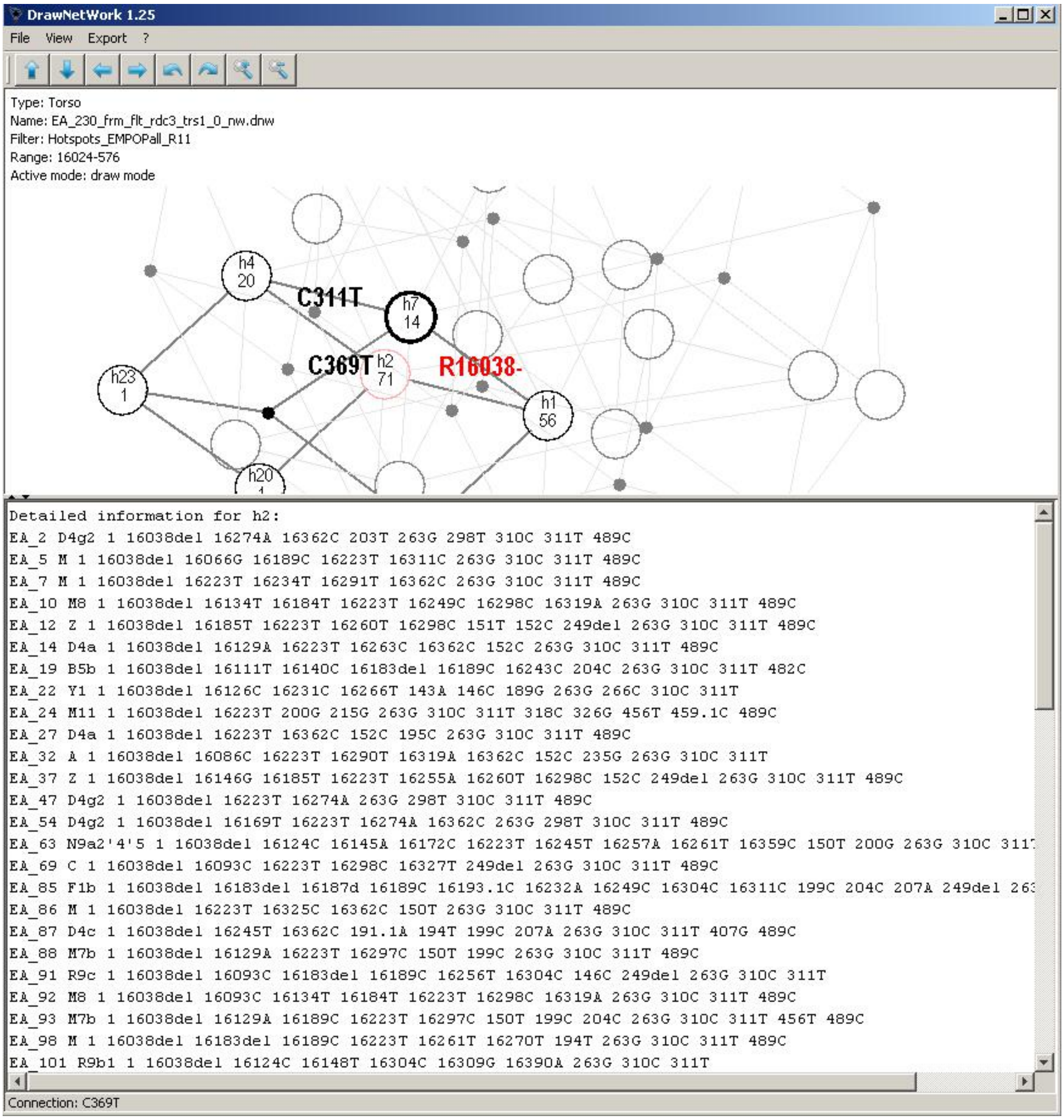

Supplement: Supplementary Figure 5 [file CroatMedJ_55_s005.pdf]
